# Large-Scale Brain Networks in Board Game Experts: Insights from a Domain-Related Task and Task-Free Resting State

**DOI:** 10.1371/journal.pone.0032532

**Published:** 2012-03-12

**Authors:** Xujun Duan, Wei Liao, Dongmei Liang, Lihua Qiu, Qing Gao, Chengyi Liu, Qiyong Gong, Huafu Chen

**Affiliations:** 1 Key Laboratory for Neuroinformation of Ministry of Education, School of Life Science and Technology, University of Electronic Science and Technology of China, Chengdu, People's Republic of China; 2 Lab of Laser Sports Medicine, South China Normal University, Guangzhou, People's Republic of China; 3 Huaxi MR Research Center, Department of Radiology, West China Hospital of Sichuan University, Chengdu, People's Republic of China; University of Granada, Spain

## Abstract

Cognitive performance relies on the coordination of large-scale networks of brain regions that are not only temporally correlated during different tasks, but also networks that show highly correlated spontaneous activity during a task-free state. Both task-related and task-free network activity has been associated with individual differences in cognitive performance. Therefore, we aimed to examine the influence of cognitive expertise on four networks associated with cognitive task performance: the default mode network (DMN) and three other cognitive networks (central-executive network, dorsal attention network, and salience network). During fMRI scanning, fifteen grandmaster and master level Chinese chess players (GM/M) and fifteen novice players carried out a Chinese chess task and a task-free resting state. Modulations of network activity during task were assessed, as well as resting-state functional connectivity of those networks. Relative to novices, GM/Ms showed a broader task-induced deactivation of DMN in the chess problem-solving task, and intrinsic functional connectivity of DMN was increased with a connectivity pattern associated with the caudate nucleus in GM/Ms. The three other cognitive networks did not exhibit any difference in task-evoked activation or intrinsic functional connectivity between the two groups. These findings demonstrate the effect of long-term learning and practice in cognitive expertise on large-scale brain networks, suggesting the important role of DMN deactivation in expert performance and enhanced functional integration of spontaneous activity within widely distributed DMN-caudate circuitry, which might better support high-level cognitive control of behavior.

## Introduction

The board game Chess involves many aspects of high level cognition and requires sophisticated problem solving skills [1], [2], and it is considered one of the most mentally taxing of pursuits [3]. During chess playing, many kinds of cognitive processes are involved, e.g. attention, visuo-spatial perception, motivation, working memory, and decision making [4], [5], [6], [7]. Brain imaging studies have suggested that the human brain is delicately organized into multiple distinct yet inherently interacted functional networks to support these processes [8], [9], [10]. For instance, a central-executive network (CEN), which includes the dorsolateral prefrontal cortex (DLPFC) and posterior parietal cortex (PPC), is critical for working memory, attentional control, and judgment and decision making in the context of goal-directed behavior [8], [11], [12]; a dorsal attention network (DAN), which includes the intraparietal sulcus (IPS) and the junction of the precentral and superior frontal sulcus (frontal eye field, FEF), plays a key role in top-down orienting of attention, visuo-spatial perception, and goal-directed stimulus response selection and action [10], [13]; and lastly, a salience network (SN), anchored by dorsal ACC and the fronto-insular cortex (FIC), is responsible for salience processing and decision making [14], [15]. During performance of cognitively demanding tasks, activation in these brain networks typically increases, while another network, the default mode network (DMN), has been consistently shown to decrease activation.

The DMN is generally thought to consist of a set of brain regions including the precuneus/posterior cingulate cortex (P/PCC), ventral ACC/medial prefrontal cortex (MPFC), angular gyrus (AG) and medial temporal cortex. It has been linked to self-referential and reflective activity that specifically includes episodic memory retrieval, inner speech, mental imaging, and the theory of mind [9], [16], [17], [18]. The repeated observation that the DMN paradoxically exhibits high levels of baseline activity at rest and decreases from this baseline across a wide range of goal-directed behaviors led to the characterization of this network as a “default mode” of brain function [19], [20], [21]. The deactivation of the DMN has been previously explained as the reallocation of cognitive resources in the brain in order to focus more on the task and suppress unrelated or irrelevant thoughts [22]. Recent studies also suggest that the magnitude of task-induced DMN deactivation increases with task difficulty [23], [24], [25]. Additionally, successful performance of attention-demanding cognitive tasks is always associated with enhanced deactivation of DMN [26], [27].

Cognitive performance relies on the coordination of the cognitive networks with the DMN; that is, increased activation in the cognitive networks and decreased activity in the DMN [8], [22], [27], [28], [29]. Within both kinds of networks, brain regions are not only temporally correlated during different tasks, but also show highly correlated spontaneous activity during a task-free state [22], [30], [31]. Since spontaneous activity is likely to reflect the history of coactivation within local networks [32], and it can also account for the variability of task-evoked responses [33], both task-evoked and task-free network activity can be related to individual task performance and previous experiences [30]. Evidence from previous imaging studies on skill learning indicates that learning and practice exhibit shifts in the set of neural structures that contribute to performance [34], [35], [36], [37]. Raichle [38] demonstrated a complex picture of widely distributed change (both increases and decreases) in the activity of brain systems associated with task performance after repeated cognitive skill learning, and highlighted the reallocation of brain resources on large systems levels. Moreover, recent studies on resting-state brain function indicate that prior experience in the form of skill learning can change the pattern of spontaneous cortical activity between different brain networks in specific way [39]. However, most of those studies focused on the influence of short-term practice of cognitive skills on brain functional circuits, and little is known about the changes in large-scale brain networks in response to long-term and extensive experience of high-level cognitive skill learning.

Cognitive experts like professional board game players represent an ideal model with which to investigate the effect of long-term skill acquisition in high-level cognitive domains on large-scale brain networks associated with cognitive function, due to the various cognitive processes that are involved in their learning and practice. Chess, as one of the most popular strategic board games, has been widely used to study individual differences in visuo-spatial perception, working memory, problem solving, and judgment and decision making [7], [40], [41], [42], [43], [44], [45]. Brain imaging studies on chess cognition indicate that frontal and posterior parietal circuits, which are known to be involved in working memory, visuo-spatial attention and perception, are engaged in chess playing [1], [4], [5], [46]. However, traditional theories of expert performance and skill acquisition indicate that superior performance levels attained after learning and practice reflects the importance of domain-specific knowledge in chess expertise, which suggests that skills do not reside in differences in short-term memory capacity or perceptual abilities, but in the number of chunks held in long-term memory [7], [41]. Ognjen Amidzic et al. found that highly skilled chess grandmasters had more bursts of gamma-band activity in the frontal and parietal cortices compared to amateur players during matches, which might be associated with the retrieval of chunks from expert memory [5]. Campitelli [40] compared a grandmaster and an international chess master with a group of novices in a memory task with chess and non-chess stimuli, and findings revealed that experts activated different brain systems from those of novices. The latest report from Wan et al. [2], who studied the neural basis of intuitive best next-move generation in Japanese chess experts, indicated that experts uniquely recruited brain activation in the precuneus-caudate circuit during quick perception of chess patterns and intuitive generation of best next-move in chess. These findings suggest that chess grandmasters may differ from novices in certain brain networks that support the process of chess performance.

The aim of the present study was to investigate the influence of long-term learning and practice of cognitive expertise over the four typical large-scale brain networks associated with cognitive behavior: the DMN and three other cognitive networks (CEN, DAN, SN). To address this issue, we assessed fifteen grandmaster and master level Chinese chess players (GM/M) and fifteen novice players, with a Chinese chess problem-solving task and a task-free resting-state experiment, by means of functional magnetic resonance imaging (fMRI). By assessing modulations of network activity during task as well as resting-state functional connectivity of those networks in groups of GM/Ms and novices, we expected to provide evidence for the effect of high-level cognitive expertise on both task-related and task-free network activity, and further provide insights into the functional reorganization and plasticity within widely distributed circuitry, in response to environmental demands with respect to high-level cognition.

## Materials and Methods

### Participants

Two groups of subjects were recruited and studied. The first group consisted of fifteen grandmaster and master level Chinese chess players (GM/M) (five female, aged 28.73±7.71 years) who had a mean period of 13.67±6.68 years of tournament and training practice and scored between 2,200 and 2,600 on Elo's chess-skill rating scale [47]. All of them were recruited from the First National Intelligence Games held in Chengdu, China. The second group consists of fifteen novice players (five female, aged 25.33±6.01 years) who knew the rules and simple strategies of playing Chinese chess. All subjects were right-handed and with no history of psychiatric or neurological disorders. Both groups were tested with Raven's Standard Progressive Matrices, and the two groups did not differ on observation skills or clear-thinking ability according to the test (p = 0.15, t = −1.49). The present study was approved by the local Institutional Review Board of the West China Hospital of Sichuan University, and informed written consent was obtained from all subjects.

### Materials

During functional magnetic resonance imaging (fMRI) scanning, subjects carried out two experiments: first a resting-sate experiment, and then a Chinese chess problem-solving task.

During the Chinese chess problem-solving task, subjects were presented with three kinds of stimuli: a blank chessboard, boards with pieces placed randomly, and patterns of Chinese chess spot game with checkmate problems.

- Blank board condition. A blank board without any pieces was used as a baseline condition. In this condition, subjects were instructed simply to look at the center of the board and to not think of anything in particular. Each board was presented for 20 s, and then a cue appeared and lasted for 2 s. During these 2 s, subjects had to randomly press one of the two buttons at hand.
- Random board condition. Pieces were positioned in a randomly dispersed pattern on the board. Each of them was placed off of the line intersections to avoid initiating subjects' thoughts of possible moves (In Chinese chess, the pieces are played on the line intersections on the board). In this condition, subjects were also instructed to look at the center of the board and to not think about anything in particular. Each random board was presented for 20 s, and then a cue appeared and lasted for 2 s. During these 2 s, subjects had to randomly press one of the two buttons at hand.
- Game condition. Checkmate problems were selected from the Chinese Chess Composition Warehouse (http://www.dpxq.com/). For each problem, the total number of pieces for each piece type (Red/Black) was ranged from 4 to 6, and the Red can beat the Black in five steps if appropriate strategies were used. In this condition, subjects were instructed to work out the strategies (a series of moves) to checkmate the Black with the Red pieces. Each board was presented for 20 s, and subjects had to solve the problem within this time. When the time was up, they had to stop thinking and indicate whether or not a solution was figured out by pressing the buttons. The cue used to notify that time was up as well as to elicit the manual response was the same as used in the Blank board condition and Random board condition. It lasted for 2 s, and subjects had to make a response within this time. All the checkmate problems were tested by other similar skill level chess experts to make sure that all of them had approximately equal complexity and 20 s was a reasonable amount of time to come up with a solution.

### Procedure

The experiment was divided into three parts: an instruction phase, a scanning phase during which subjects performed the task, and a post-scan debriefing session. In the instruction phase, the subjects were familiarized with the three kinds of stimuli and given a number of practice trials. The stimuli of the Game condition used for practice were different from those used in the scanning phase.

The scanning phase of the experiment was divided into two sessions. The first session was a 410 s resting-state run, during which subjects were instructed to relax with their eyes open and visual fixation on a hair-cross centered in the screen. The second session was a block-design Chinese chess problem-solving task run. Each individual presentation of the three kinds of stimuli constituted one 22 s block of a block-design paradigm. Each block displayed the sequence of blank, random and game conditions, and repeated 9 times in the same order, but each time with new stimuli in both the random and game conditions. At the start of the task run, the screen remained black for 10 s to allow time for magnetization to reach steady state, and the total scan time of this run was 604 s.

To ensure that subjects remained actively engaged in the chess problem-solving task, after scanning, they were required to say aloud their strategy in a randomly selected checkmate problem they had successfully worked out during the scanning. No subjects failed to report their checkmate strategy used in a successfully worked out problem.

### Data Acquisition and Preprocessing

Scanning was performed on a 3T Siemens Trio system (Erlangen, German) at the MR Research Center of West China Hospital of Sichuan University, Chengdu, China. T2-weighted fMRI images were obtained via a gradient-echo echo-planar pulse sequence (TR, 2000 ms; TE, 30 ms; flip angle = 90°; whole head: 30 axial slice, each 5 mm thick (without gap); voxel size = 3.75
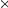
3.75
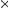
5 mm). The resting-state run contained 205 image volumes, and the task contained 302 volumes. The first five volumes of each run were discarded for magnetization stabilization.

fMRI images were preprocessed using Statistical Parametric Mapping-8 (SPM8, Wellcome Trust Centre for Neuroimaging, London, UK. http://www.fil.ion.ucl.ac.uk/spm), with the same procedure and parameters for both the resting state experiment and the task condition. Spatial transformation, which included realignment and normalization, was performed using three-dimensional rigid body registration to correct for head motion. The realigned images were spatially normalized into a standard stereotaxic space at 2
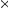
2
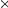
2 mm, using the Montreal Neurological Institute (MNI) echo-planar imaging (EPI) template. A spatial smoothing filter was employed for each brain's three-dimensional volume by convolution with an isotropic Gaussian kernel (FWHM = 8 mm) to increase the MR signal-to-noise ratio. Then, for the fMRI time series of the task condition, a high-pass filter with a cut-off of 1/128 Hz was used to remove low-frequency noise. The resting state fMRI time series underwent a temporal bandpass filter (0.01–0.08 Hz), and then several sources of spurious variance, along with their temporal derivatives, were removed from the data through linear regression: six parameters obtained by rigid body correction of head motion, the whole-brain averaged signal, signal from a ventricular region of interest, and signal from a region centered in the white matter. This regression procedure removed fluctuations unlikely to be involved in specific regional correlations.

### General Linear Model Analysis

For the Chinese chess problem-solving task, statistical analysis was performed using the general linear model (GLM) and the theory of Gaussian random fields [48], [49], as implemented in SPM8. Subject-specific regressors of interest were assembled by convolving delta functions (corresponding to the entire block length of each block for each condition) with a canonical hemodynamic response function (HRF). Parameter estimates were used to calculate the appropriate linear contrast. To detect the activated areas involved in the problem-solving process of chess playing, the game condition was contrasted both against the blank board condition and random condition for each subject. To extend inference based on individual statistical analyses, a random-effect analysis was performed for groups of GM/Ms and novices by using one sample *t*-test in SPM8, respectively. The significance level for each group was set at *p*<0.05 using the AlphaSim correction (a combination of threshold of *p*<0.001 and a minimum cluster size of 22 voxels). This correction was conducted using the AlphaSim program in the REST software (http://www.restfmri.net), which applied the Monte Carlo simulation to calculate the probability of false positive detection by taking both the individual voxel probability thresholding and cluster size into consideration [50].

To further test the activation differences between GM/Ms and novices when they performed the Chinese Chess task, the statistical parametric maps of the GM/M group were compared to those of the novice group using two-sample *t* tests (*p*<0.05, AlphaSim corrected). The group comparison was restricted to the voxels with significant activation/deactivation maps of either GM/Ms or novices by using an explicit mask from the union set of the one-sample *t* test results (p<0.05, AlphaSim corrected) of the two groups, respectively for activation and deactivation.

### Resting-State Functional Connectivity Analysis

As expected, the Chinese chess problem-solving task evoked increased brain activation in the cognitive networks which mainly including CEN, DAN and SN, as well as deactivation of DMN. For each network, four regions of interest (ROI) which were identified by peak foci in canonical regions on the activation map, and were treated as seeds in the resting-state functional connectivity analysis.

A reference time series for each seed was obtained by averaging the fMRI time series for 27 adjacent voxels with the peak foci at the center. Correlation maps were produced by computing the correlation coefficient between the Blood Oxygenation Level Dependent (BOLD) time course, extracting from a seed region, and the time course from all other brain voxels. Coefficients were converted to a normal distribution by Fischer's *z* transformation. Population-based *z*-score maps for the four seeds in each network were combined by using a conjunction analysis. Voxels were included in the conjunction map only if they were significantly correlated with three of the four seed regions. Then *z* score maps were combined across subjects by using a random-effects analysis for groups of GM/Ms and novices using a one-sample *t*-test in SPM8, respectively. The significance level for each group was set at *p*<0.05, AlphaSim corrected.

Subsequently, the *z*-score maps in GM/Ms were compared to those in novices using two-sample *t*-tests (*p*<0.05, AlphaSim corrected). The group comparison was restricted (masked) to the voxels with significant positive correlation maps of either GM/Ms or novices.

## Results

### Behavioral Results

As expected, GM/Ms performed significantly better than novice players (*t* = 9.12, *p*<10^−8^) in the game condition of the Chinese chess problem-solving task, successfully come up with a solution in 7.8 boards (*SD* = 1.21), while novices successfully worked out 2.27 boards (*SD* = 2.02). To further examine the differences between GM/Ms and novices on general intelligence, both groups were tested by Raven's Standard Progressive Matrices, a widely used intelligence test of abstract reasoning, and two groups did not differ on observation skills and clear-thinking ability according to the test (*p* = 0.16, *t* = −1.50).

### Task-Evoked Activation of CEN, DAN and SN, and Deactivation of DMN

**Figure 1:**
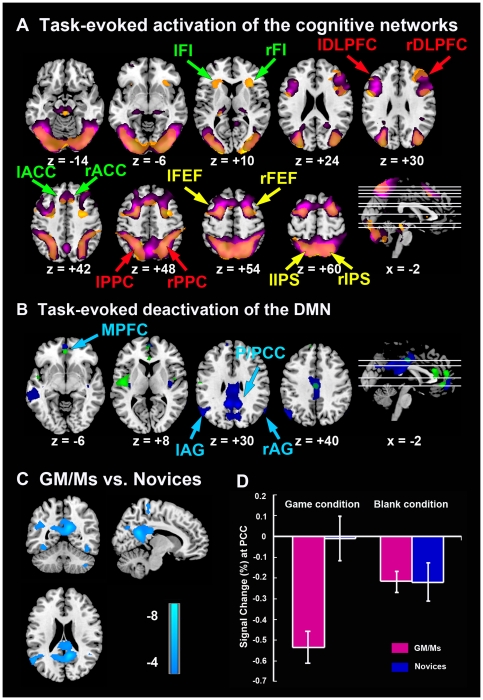
Activation in the cognitive networks and deactivation in the DMN during Chinese chess problem-solving task (Game condition vs. Blank board condition). (A) General Linear Model (GLM) analysis revealed regional activations for GM/Ms (orange) and novices (purple) in the cognitive networks, which included bilateral DLPFC and PPC in the CEN (indicated by red arrow), FEF and IPS in DAN (yellow arrow) and FIC and ACC in the SN (light green arrow) (*p*<0.05, AlphaSim corrected). (B) Deactivation map for GM/Ms (blue) and novices (light green) in PCC, MPFC and bilateral AG, which constitutes the DMN (light blue arrow) (*p*<0.05, AlphaSim corrected). (C) PCC, left AG and middle temporal gyrus which located in the DMN shows significantly greater deactivation during Chinese chess problem-solving task in GM/Ms than in novices (*p*<0.05, AlphaSim corrected). (D) Percent signal change at the PCC (MNI coordinate: 10, −52, 28) during game condition and blank board condition. Error bars represent standard error of the mean for each column.

As reported previously [1], we found significant activation of the frontal and parietal cortices during the Chinese chess problem-solving task (game condition vs. blank board condition) in both groups of GM/Ms and novices, which mainly included DLPFC, ACC, PPC, IPS, FEF and FIC (Fig. 1A, Table 1). These regions are all consistent with the pattern of cognitive networks, and we extend this finding to characterize network-specific responses in the CEN, DAN and SN, which were known as canonical sub-networks of the cognitive network. Moreover. both groups demonstrated significant deactivation in the MPFC, while additional robust P/PCC, AG, and middle temporal gyrus deactivation were found in GM/Ms (Fig. 1B, see also Table 1). This pattern of activity included all the areas typically thought to be part of the DMN, which has been consistently shown to deactivate during cognitively demanding tasks that evoke activation in the cognitive networks. Similar findings from contrasts between the game condition and random condition are described in (Fig. S1, Table S1).

**Table 1:** Coordinates of CEN, DAN, SN activation and DMN deactivation during Chinese chess problem-solving task (Game condition vs. Blank board condition) (*p*<0.05, AlphaSim corrected).

Regions	R/L	BA	Peak-MNI coordinates	*t*-score	BA	Peak-MNI coordinates	*t*-score
		*GM/M*			*Novice*		
**CEN**							
DLPFC	L	9	−48, 34, 34	4.59	9	−40, 22, 28	8.25 †
	R	46	48, 40, 30	5.55	9	46, 34, 24	6.95 †
PPC	L	40	−36, −44, 46	6.30	40	−40, −40, 42	7.91 †
	R	40	42, −42, 48	7.40 †	40	46, −36, 48	7.27
**DAN**							
IPS	L	7	−20, −60, 54	11.04 †	7	−26, −60, 54	9.50
	R	7	20, −60, 52	9.11	7	20, −62, 60	11.52 †
FEF	L	6	−26, 2, 52	12.10	6	−26, 8, 60	13.39 †
	R	6	26, 6, 54	10.20 †	6	26, 14, 60	9.97
**SN**							
FIC	L	45	−32, 26, 12	5.25 †	/	/	/
	R	13	34, 22, 10	6.15 †	47	30, 24, −2	4.10
ACC	L	32	−6, 22, 40	4.76	6	−8, 24, 40	5.32 †
	R	32	8, 22, 42	5.85	6	10, 28, 42	5.88 †
**DMN**							
PCC	L	31	−6, −48, 32	−8.83	/	/	/
	R	31	10, −52, 28	−9.16 †	/	/	/
VMPFC	L/R	10	−2, 54, −6	−6.33	9	−2, 52, 18	−6.51 †
AG	L	39	−44, −60, 32	−5.59 †	/	/	/
	R	40	60, −62, 28	−6.42 †	/	/	/

Abbreviation: BA, brodmann area; R/L, right or left; CEN, central-executive network; DLPFC, dorsolateral prefrontal cortex; PPC, posterior parietal cortex; DAN, dorsal attention network; IPS, intraparietal sulcus; FEF, Frontal Eye Field; SN, salience network; FIC, fronto-insular cortex; ACC, anterior cingulate cortex; DMN, default mode network; PCC, posterior cingulate cortex; VMPFC, ventromedial prefrontal cortex; AG, angular gyrus. Regions labeled by † were used as seeds in the subsequent resting-state functional connectivity analysis to construct unbiased correlation maps for CEN, DAN, SN and DMN.

### Between-Group Comparisons in Task-Evoked cognitive networks and DMN

To compare differences in activation/deactivation of cognitive networks and the DMN between groups of GM/Ms and novices, map-wise comparisons of the game condition greater than blank board condition were performed.

First, whole-brain map-wise between-group comparisons in activation did not demonstrate any marked difference between groups of GM/Ms and novices in the brain regions anchored in the cognitive networks including CEN, DAN and SN, when contrasting the game condition to both the blank board condition and random condition.

Second, we compared the deactivation differences between the two groups during the game condition versus blank board condition. Relative to novices, GM/Ms showed significantly broader deactivation in the PCC, AG and the middle temporal gyrus (*p*<0.05, AlphaSim corrected), all areas that are typically considered important parts of the DMN, thus suggesting a more extensive suppression of DMN activity in GM/Ms during the chess problem-solving task (Fig. 1C, Table 2). To illustrate the negative BOLD response in the default mode network, we plotted the signal change at PCC (MNI coordinate: 10, −52, 28) both during game condition and blank board condition (Fig. 1D). Fig. 1D demonstrates that GM/Ms exhibited greater deactivation (i.e. a greater magnitude of below-baseline BOLD signal changes) during game condition than novices (*p* = 0.0003, *t*(28) = −4.17). Furthermore, there's no difference for the blank board control condition between the two groups (*p* = 0.97, *t*(28) = 0.035). A between-group deactivation comparison of the game condition versus random condition also revealed significantly broader deactivation in PCC and AG in GM/Ms than novices (For more detail, see Table S2 and Fig. S1(C)).

**Table 2:** Deactivation differences in the DMN during Chinese chess problem-solving task (Game condition vs. Blank condition) (Two sample *t*-test, *p*<0.05, AlphaSim corrected).

*GM/M vs. Novice*				
Regions	R/L	BA	Peak-MNI coordinates	*t*-score
Posterior Cingulate Cortex	L	23	−4, −52, 22	−4.93
	R	/	10, −52, 22	−6.84
Angular Gyrus	L	/	−52, −60, 24	−4.99
Middle Temporal Gyrus	L	/	−58, −38, −6	−4.36

### Between-Group Comparisons of Resting-State Functional Connectivity in the cognitive networks and the DMN

To assess the effect of cognitive expertise on resting-state functional connectivity of the cognitive networks and the DMN in GM/Ms, we chose 16 regions as seeds (bilateral DLPFC and PPC for CEN; bilateral IPS and FEF for DAN; bilateral FIC and ACC for SN; right PCC, left VMPFC and bilateral AG for DMN), each of which was identified by peak foci in canonical regions on the activation/deactivation map of task (Table 1, Fig. 1). To construct unbiased resting-state functional connectivity maps, conjunction analysis was used to combine the correlation maps of the four seeds in each network. Voxels were included in the conjunction map of each network only if they were significantly correlated with three of the four seed regions.

**Figure 2:**
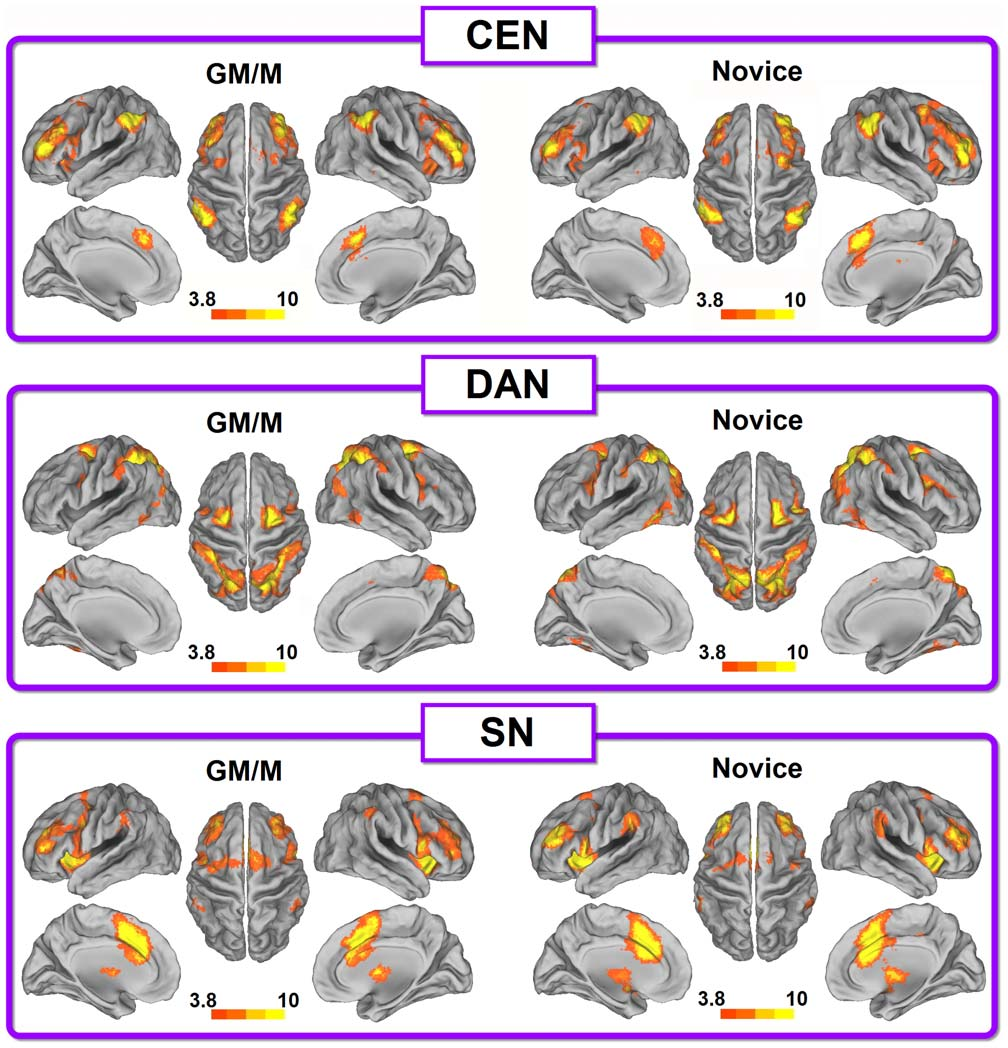
The population-averaged correlation maps of CEN, DAN and SN in GM/Ms and novices during resting-state experiment (One-sample *t*-test, *p*<0.05, AlphaSim corrected). Seeds for resting-state functional connectivity calculation of these three networks were labeled by † in Table 1.

The population-averaged correlation maps were generated for each of the four networks and each of the two groups by using random effects analysis across the population. The main patterns of CEN, DAN and SN activations were equivalent in the two groups (Fig. 2. One-sample *t*-test, *p*<0.05, AlphaSim corrected). When the population-based correlation maps for those three networks of the two groups were entered into a two-sample *t*-test, no significantly differences were detected between the two groups.

**Figure 3:**
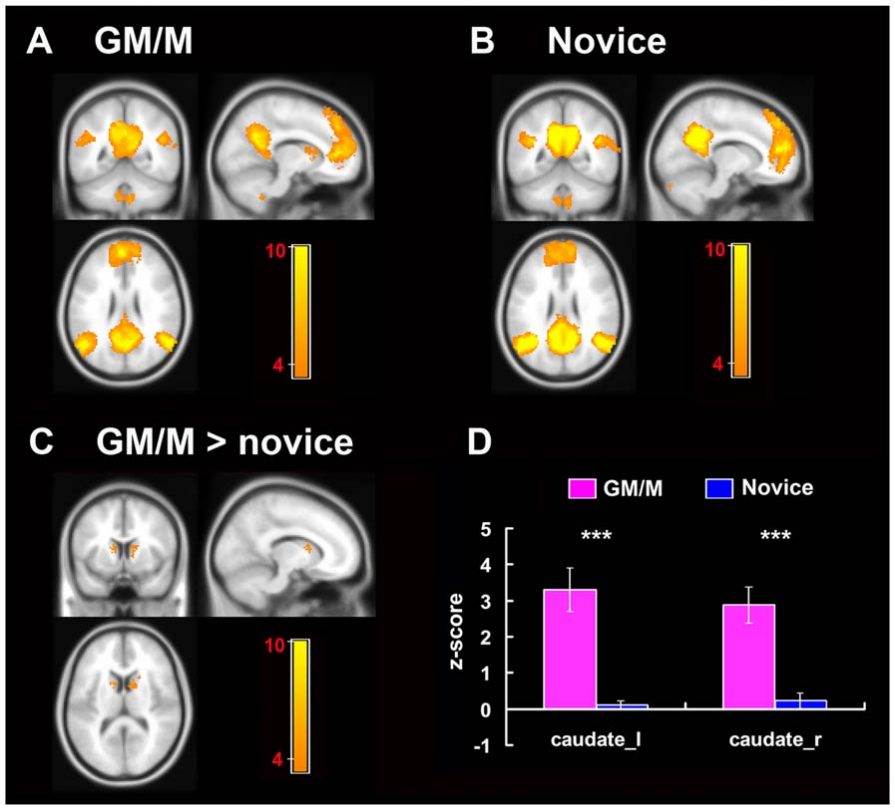
Results of the resting-state functional connectivity in the DMN. A and B demonstrate the population-averaged correlation maps of the DMN in GM/Ms (A) and novices (B) during resting-state experiment (One-sample *t*-test, *p*<0.05, AlphaSim corrected). Seeds for resting-state functional connectivity calculation of the DMN were labeled by † in Table 1. (C) Between-group comparisons reveal significantly increased connectivity of DMN with the caudate nucleus in GM/Ms relative to novices (Two sample *t*-test, *p*<0.05, AlphaSim corrected. Peak MNI coordinates x, y, z: left caudate −12, 8, 12; right caudate 8, 10, 10). (D) Plots of the *z*-score in the bilateral caudate in the population-averaged correlation maps of DMN in GM/Ms (purple) and novices (blue).

The main pattern of DMN correlation maps of the two groups were approximately similar, with the inclusion of regions in the PCC, bilateral AG, ventral and dorsal MPFC, inferior temporal cortex, medial temporal cortex and medial cerebellum (Fig. 3A, B. One-sample *t*-test, *p*<0.05, AlphaSim corrected). When the population-based DMN correlation maps of the two groups were entered into a two-sample *t* test, a significantly increased positive correlation was found in GM/Ms relative to novices in the caudate nucleus (Fig. 3C. two-sample *t* tests, *p*<0.05, AlphaSim corrected), a region not typically thought to be part of the DMN. No brain region was found to have marked increased functional connectivity in DMN in novices than in GM/Ms.

## Discussion

In this study, we compared the DMN and three other cognitive networks over the Chinese chess problem-solving task and a resting-state experiment in groups of GM/Ms and novices to assess the effect of long-term and intensive practice of high-level cognitive skills on large-scale brain networks. There were three main findings. Firstly, the Chinese chess problem-solving task evoked the expected modulations of activity in the DMN and the cognitive networks which included the CEN, DAN, and SN. That is, there was activation in the cognitive networks and deactivation in the DMN. Secondly, relative to novices, GM/Ms showed a more extensive disengagement of the DMN in the chess problem-solving task, which mainly displays on broader task-related decreases in the precuneus/posterior cingulate cortex (P/PCC) and the angular gyrus; when using these deactivated DMN regions as seeds in resting-state functional connectivity analysis, we found that, in the group of GM/Ms, the DMN was increased with a connectivity pattern associated with the caudate nucleus, a region involved in decision-making and motivational processes but not typically thought to be part of the DMN [51], [52], [53], [54]. Thirdly, there was no significant difference in the task-induced activation or task-free functional connectivity within the cognitive networks between two groups.

### Modulations of brain activity in the cognitive networks and the DMN during Chinese chess problem-solving task

In both groups of GM/Ms and novices, we identified the existence of activation in the cognitive networks and deactivation in the DMN during the Chinese chess problem-solving task. The brain activity in the cognitive network was characterized to subnetwork-specific responses in the CEN, DAN and SN, as identified by the major coactivated nodes thought to comprise these networks in the activation map [8], [11], [13], [55]. These findings were consistent with previous brain imaging studies across several task domains that, during a number of goal-directed cognitive-demanding tasks, the CEN, DAN, and SN typically show increases in activation. The activation patterns observed in these three networks during the chess problem-solving task in the present study were largely consistent with those mapped from other previous specific task-induced activation [8], [13], [56]. Specifically, the dorsolateral part of the prefrontal cortex (DLPFC) and the posterior parietal cortex (PPC), which constitute an important part of the CEN, are critical for working memory and goal-directed stimulus-response selection and action [8], [11], [12]; the intraparietal sulcus (IPS) and the frontal eye field (FEF) anchored in the DAN are involved in voluntary (top-down) orienting and show activity increases after presentation of cues indicating where, when, or what subjects should direct their attention to; The fronto-insular cortex (FIC), a key node of the SN, is associated with interoceptive awareness and subjective salience, and the anterior cingulate cortex (ACC) anchored within the SN is involved in error monitoring and response selection [8], [14], [15]. The SN further has an important role in cognitive control related to switching between the DMN and those task-positive cognitive networks [56]. These nodes of the cognitive networks (CEN, DAN, SN) have consistently shown to be activated during cognitively demanding tasks to support the processes of attention, visuo-spatial perception, motivation, execution, working memory, and decision making, while the DMN, whose primary role may be to support internally oriental mental processes [57], typically shows decreased activity, reflecting the reallocation of cognitive resources from task-irrelevant mental processes to focus more on the task at hand [21], [25], [26], [58].

Chess has long served as a model task environment for research into psychological processes, such as perception, memory and problem solving [7], [59]. To fully elicit the brain activation associated with various cognitive processes in chess performance, we conducted a chess problem-solving experiment on each subject, in which subjects were instructed to work out the strategies (to find a series of moves) leading to a checkmate within a period of relatively sufficient time. To accomplish the task, subjects have to activate a deliberative search processing that evaluated a candidate move in terms of potential future positions reached via a branching tree of available moves for the two sides [2], [60]. This kind of information processing involves high-level cognition mainly including top-down orientating of attention, visuo-spatial perception of board pattern, and error monitoring of judgment and response selection, which are considered to be potentially evoked activations of cognitive networks like the CEN, DAN and SN, as well as deactivation in the DMN. The modulation of brain activity in large-scale brain networks during chess performance has not been directly assessed before, and what's more, to our knowledge, this is the first time that the deactivation of the DMN during chess problem solving has been reported, thus adding to a growing literature that goal-directed cognitive behavior increases activity in brain networks whose function supports task execution and decreases activity in the network supporting unrelated or irrelevant internal-oriented processes.

### The influence of cognitive expertise on the DMN both in task-induced deactivation and task-free functional connectivity

Both the GM/Ms and novices exhibited increased activity in the CEN, DAN, and SN, and decreased activity in the DMN during performance of the chess problem-solving task while comparing the game condition to both the blank board and random conditions. Interestingly, between-group comparisons of the task-induced deactivation revealed that GM/Ms had a more extensive disengagement of the DMN than novices, demonstrating much more robust task-induced deactivation during the game condition. Since the DMN has consistently shown to be active during periods when a person is awake but not engaged in a specific cognitive task [26], it has been suggested that task-induced deactivation of the DMN reflects the reallocation of cognitive resources from task-irrelevant processes that occur during the conscious resting state to task-relevant processes required during the execution of an active task [21], [61]. One possible reason why GM/Ms exhibited a stronger deactivation effect in the DMN is that, in the very beginning of exposure to a new position, GM/Ms were more likely to fixate on relevant squares, and did so more quickly to encode chess information than novices [7]. Hence, they could quickly and fully concentrate their attention to solve the problems, thus suppressing irrelevant internally-directed thoughts. This kind of suppression allows reallocation of cognitive resources from task-irrelevant processes to task-relevant processes, and results in robust deactivation of the DMN. Similar evidence has also been found by Brefczynski-Lewis and colleagues, showing that expert meditators had less involvement in the default-mode regions related to task-irrelevant thoughts, since they had less of a reaction to the distractions than novice meditators [62]. Another possibility is that chess experts engaged in deeper processing during the deliberate search of the chess problem-solving task. During this process, players have to examine branches step by step, starting from a promising next move to check whether checkmate could be reached regardless of the opponent's moves; if not, they moved the search to branches starting from another promising next move [2]. The deeper the player searches, the higher is the cognitive load for the player, leading to an increasing of the deactivation magnitude in the default mode regions. Multiple studies have demonstrated the ‘beneficial’ deactivation in the DMN during successful performance of cognitive tasks, consistent with the hypothesis of behavioral competition between task-focused attention and processes subserving stimulus-independent thought [21], [22]. The patterns of deactivation that we observed in GM/Ms are consistent with the above idea that successful performance of goal-directed cognitive tasks needs the coordination of both the cognitive networks and the DMN, which was reflected in the behavioral result that GM/Ms performed significantly better than novice players in the game condition of the Chinese chess problem-solving task.

Recently, assessments of brain functional connectivity were conducted in order to investigate the level of integration of brain systems at a resting state when no task was performed [9]. Task-free spontaneous neural activity has been proposed to play an important part in maintaining the ongoing representations of conscious activity in the resting brain, and it demonstrates temporal coherence between brain regions that are anatomically connected and functionally related through co-activity in response to task performance [63], [64]. Additionally spontaneous coherence in cortical networks also reflects individual differences in cognitive performance and learning experiences [39], [65], [66]. Since difference was found in the DMN during the chess problem-solving task, we wondered whether the difference was task-specific, or task-independent, intrinsically existed between the two groups. By using the task-induced deactivated DMN regions as seeds in resting-state functional connectivity analysis, we found that functional connectivity of the DMN in GM/Ms was increased with a connectivity pattern associated with the caudate nucleus, a region involved in decision-making and motivational processes but not typically thought to be part of the DMN [51], [52], [53], [54].

The caudate nucleus is part of the dorsal striatum, which is considered to be involved in a wide range of functions including motor, motivational, cognitive control and reward processing [51], [53], [67], [68]. In particular, the caudate nucleus is thought to be responsible for the formation of stimulus-response association, which mediates goal-directed action [69], [70], [71], [72]. More relevantly, Wan et al. [2] found that the caudate was recruited during the intuitive generation of best next-move of Japanese chess checkmate problems in professional players. In chess experts, chunks of pieces are associated with the best next-move in players' long-term memory. Thus, the perception of chunks automatically evokes the idea of the best next move [2], [41]. This idea is similar to that of stimulus-action association which involves the caudate nucleus [2]. Wan et al. also found that activation in the precuneus of the parietal lobe was significantly associated with quick perception of board patterns, suggesting the important role of the precuneus-caudate circuit in chess expertise. Since the precuneus is considered to be a vital node of the DMN, long-term and frequent engagement of the precuneus-caudate circuit during chess training might account for the increased connectivity of the striatal-DMN loop in the brain of GM/Ms, based on the previous evidence that the history of activation changes spontaneous connectivity [39].

However, in the present study, there was no activation in the caudate nucleus during the problem-solving task. This is consistent with the finding of Wan et al. that the caudate is only recruited during quick generation of the next move, but not activated during deliberative search. The major process in the present chess problem-solving task was deliberative search, during which subjects examined branches step by step to determine whether checkmate could be reached. We used this paradigm to elicit the activation of funcitonal networks associated with top-down attention, executive action and salience processing, as well as the accompanying deactivation of DMN.

### Persistent cognitive networks in GM/Ms both during task and rest

Although the two groups showed learning-related differences in the DMN both during task and rest, no difference was found in the three cognitive networks. Activity in the CEN, DAN and SN are associated with top-down modulation of attention and working memory, which supports task execution. Previous studies on patients and aging people indicated that failure to deactivate the DMN might due to the supplement of activation in the cognitive networks, i.e. extensive activation in the networks [26], [73]. However, in the present study, subjects were all healthy adults with no history of psychiatric or neurological disorders or cognitive impairments, and were able to fully focus on goal-directed cognitive actions, thus exhibiting regular activation patterns of cognitive networks during a chess problem-solving task. Moreover, this result is also consistent with the recent finding that professional and amateur players share the same activation pattern during deliberative search process in chess [2].

### Consistency of task modulation and resting-state functional connectivity of large-scale networks in GM/Ms

The aim of the present study was to investigate the differences of four large-scale brain networks between GM/Ms and novices during a chess problem-solving task and resting state. By comparing the four networks between the two groups, we found that the CEN, DAN and SN had no significant difference neither in task-induced activation nor in resting-state functional connectivity. However, with respect to the DMN, both task-induced deactivation and resting-state functional connectivity exhibited significant differences between the two groups, demonstrating the influence of long-term learning and practice of cognitive expertise on the DMN.

Task-evoked activation analysis revealed deactivation differences in the DMN between the two groups, however, the differences of the resting-state functional connectivity of the DMN did not anchored within the typical DMN regions, but in the caudate nucleus (i.e. increased connectivity between the DMN and the caudate). One possible explanation of this finding maybe the differences in neurophysiological aspects measured by univariate task-based analysis and resting-state connectivity analysis. More specifically, the task-based analysis measures the BOLD signal change related to the experimental paradigm in each voxel, and the resting-state functional connectivity analysis measures the correlation strength between the seed region and non-seed brain voxels during resting-state condition. Therefore it is likely that task-dependent activation in one particular voxel might not be associated with the functional correlation between that voxel and the seed. Accordingly, differences of task-evoked deactivation in the DMN between the GM/Ms and novices might not be necessarily related to resting-state functional connectivity changes among the DMN regions, but changes between the DMN regions and other related regions (in this case, the caudate). However, further study is needed, to systematically address the question of whether increased intrinsic functional connectivity of the DMN-caudate loop leads to more deactivation of the DMN during chess problem-solving task in GM/Ms.

### Conclusion

In summary, we found that both GM/Ms and novices modulate activity in the cognitive networks and the DMN in expected ways during Chinese chess problem-solving task, adding to a growing literature that cognitively demanding tasks evoke increased activity in the cognitive networks and reduced activity in the DMN. However, relative to novices, GM/Ms showed a much more robust suppression of the DMN in the chess problem-solving task, which might facilitate successful performance. In addition, examination of resting-state functional connectivity in the DMN revealed that, compared to novices, the DMN in GM/Ms showed an increased connectivity pattern associated with the caudate nucleus, suggesting the important role of the DMN-caudate loop in chess expertise. This finding indicates that long-term and extensive experience with high-level cognitive skills can enhance functional integration within widely distributed circuitry, and this kind of enhancement in turn facilitates the communication within the network and benefits successful performance in domain-related tasks. Unlike the DMN, the cognitive networks which include the CEN, DAN and SN did not exhibit any difference in task-evoked activation or task-free functional connectivity between groups of GM/Ms and novices. Taken together, these findings demonstrate the effect of long-term training of cognitive expertise on brain activity in both domain-specific tasks and resting-state spontaneous fluctuations. It suggests the important role of the DMN deactivation in expert performance and provides further evidence for neural plasticity in intrinsic connectivity networks. That is, learning and practice can enhance functional integration within widely distributed circuitry to better support high-level cognitive control of behavior.

## Supporting Information

Figure S1 Activation in the cognitive networks and deactivation in the DMN during Chinese chess problem-solving task (Game condition vs. Random condition) in GM/Ms (A) and novices (B) (One-sample *t* test, *p*<0.05, corrected for multiple comparison). C. Between-group comparison revealed deactivation differences in the PCC and left AG of the DMN when contrast Game condition to Random condition (Two sample *t*-test, *p*<0.05, corrected for multiple comparison).
(TIF)

Table S1 Coordinates of CEN, DAN, SN and DMN activation and deactivation during Chinese chess problem-solving task (Game condition vs. Random condition) (*p*<0.05, corrected for multiple comparison).
(DOCX)

Table S2 Deactivation differences in the DMN during Chinese chess problem-solving task (Game condition vs. Random condition) (*p*<0.05, corrected for multiple comparison).
(DOCX)
