# Supplementary figures and images for: Large-Scale Brain Networks in Board Game Experts: Insights from a Domain-Related Task and Task-Free Resting State
Source: PLoS One. 2012 Mar 12;7(3):e32532. doi: 10.1371/journal.pone.0032532 (PMC3299676; doi:10.1371/journal.pone.0032532)

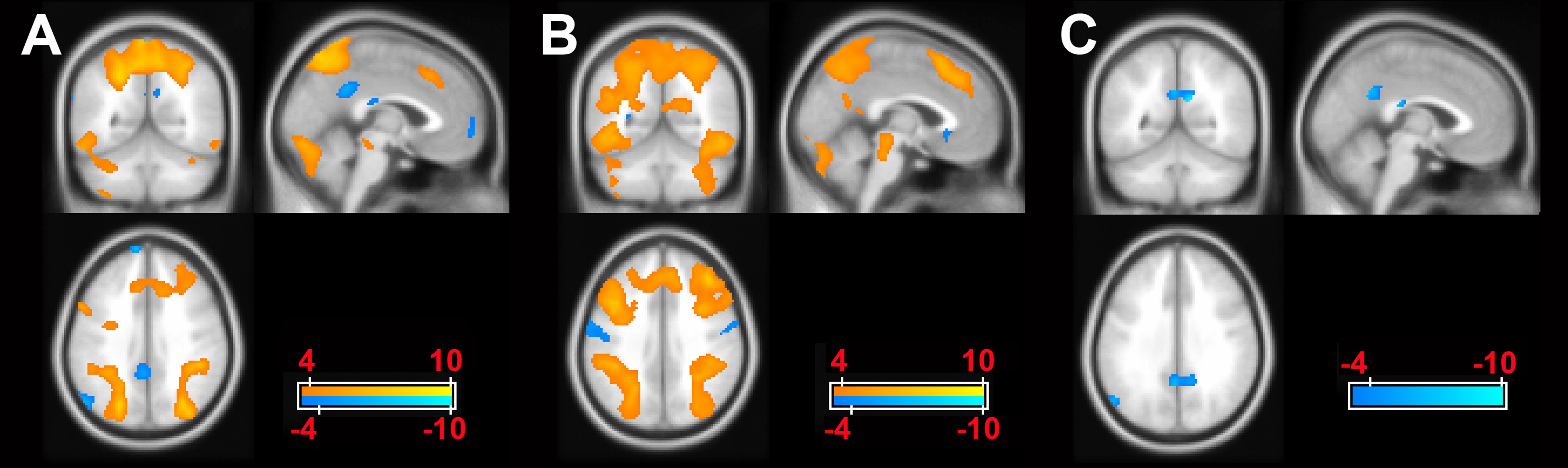

Supplement: Figure S1 — Activation in the cognitive networks and deactivation in the DMN during Chinese chess problem-solving task (Game condition vs. Random condition) in GM/Ms (A) and novices (B) (One-sample t test, p<0.05, corrected for multiple comparison). C. Between-group comparison revealed deactivation differences in the PCC and left AG of the DMN when contrast Game condition to Random condition (Two sample t-test, p<0.05, corrected for multiple comparison). (TIF) [file pone.0032532.s001.tif]
