# Supplementary material for: Large-Scale Brain Networks in Board Game Experts: Insights from a Domain-Related Task and Task-Free Resting State
Source: PLoS One. 2012 Mar 12;7(3):e32532. doi: 10.1371/journal.pone.0032532 (PMC3299676; doi:10.1371/journal.pone.0032532)
Supplement: Table S1 — Coordinates of CEN, DAN, SN and DMN activation and deactivation during Chinese chess problem-solving task (Game condition vs. Random condition) (p<0.05, corrected for multiple comparison). (DOCX) [file pone.0032532.s002.docx]

**Supplementary Information**

**Table S1. Coordinates of CEN, DAN, SN and DMN activation and deactivation during Chinese chess problem-solving task (Game condition vs. Random condition) (*p* < 0.05, corrected for multiple comparison)**

| Regions | R/L | BA | Peak-MNI coordinates | *t*-score | BA | Peak-MNI coordinates | *t*-score |
| --- | --- | --- | --- | --- | --- | --- | --- |
|  |  | ***GM/M*** | | | ***Novice*** | | |
| **CEN** | | | | | | | |
| DLPFC | L | 46 | -50, 34, 32 | 4.21 | 9 | -40, 30, 34 | 5.34 |
|  | R | 9 | 32, 30, 30 | 5.77 | 46 | 38, 34, 34 | 7.64 |
| PPC | L | 40 | -40, -48, 46 | 4.99 | 40 | -44, -44, 42 | 5.06 |
|  | R | 40 | 44, -42, 48 | 7.12 | 40 | 44, -38, 46 | 5.62 |
| **DAN** | | | | | | | |
| IPS | L | 7 | -22, -58, 48 | 7.40 | 7 | -26, -58, 50 | 6.10 |
|  | R | 7 | 26, -62, 52 | 7.38 | 40 | 34, -46, 50 | 6.91 |
| FEF | L | 6 | -28, 4, 56 | 9.19 | 6 | -28, 6, 66 | 7.86 |
|  | R | 6 | 24, 8, 56 | 9.36 | 6 | 38, 8, 62 | 5.87 |
| **SN** | | | | | | | |
| FIC | L | 45 | -32, 24, 6 | 4.76 | / | / | / |
|  | R | 47 | 34, 26, 2 | 4.59 | 47 | 32, 24, -2 | 4.15 |
| ACC | L | 32 | -8, 22, 44 | 6.37 | 6 | -6, 26, 42 | 5.71 |
|  | R | 32 | 12, 24, 40 | 5.58 | 6 | 8, 28, 40 | 5.40 |
| **DMN** | | | | | | | |
| PCC | L | 31 | -6, -48, 32 | -6.54 | / | / | / |
|  | R | 31 | 10, -52, 30 | -5.96 | / | / | / |
| MPFC/  vACC | L/R | 10 | -2, 58, -4 | -4.42 | 24 | 2, 24, -8 | -3.99 |
| AG | L | 39 | -46, -70, 42 | -5.06 | / | / | / |

Abbreviation: BA, brodmann area; R/L, right or left; CEN, central-executive network; DLPFC, dorsolateral prefrontal cortex; PPC, posterior parietal cortex; DAN, dorsal attention network; IPS, intraparietal sulcus; FEF, Frontal Eye Field; SN, salience network; FIC, fronto-insular cortex; ACC, anterior cingulate cortex; DMN, default mode network; PCC, posterior cingulate cortex; MPFC, medial prefrontal cortex; vACC, ventral anterior cingulate cortex; AG, angular gyrus.
