# Supplementary material for: Large-Scale Brain Networks in Board Game Experts: Insights from a Domain-Related Task and Task-Free Resting State
Source: PLoS One. 2012 Mar 12;7(3):e32532. doi: 10.1371/journal.pone.0032532 (PMC3299676; doi:10.1371/journal.pone.0032532)
Supplement: Table S2 — Deactivation differences in the DMN during Chinese chess problem-solving task (Game condition vs. Random condition) (p<0.05, corrected for multiple comparison). (DOCX) [file pone.0032532.s003.docx]

**Supplementary Information**

**Table S2. Deactivation differences in the DMN during Chinese chess problem-solving task (Game condition vs. Random condition) (*p* < 0.05, corrected for multiple comparison)**

| ***GM/M vs. Novice*** | | | | |
| --- | --- | --- | --- | --- |
| Regions | R/L | BA | Peak-MNI coordinates | *t*-score |
| Posterior Cingulate Cortex | L | 31 | -2, -54, 32 | -4.48 |
|  | R | / | 10, -52, 24 | -5.86 |
| Angular Gyrus | L | 39 | -54, -70, 34 | -5.62 |
